# LncRNA Riken‐201 and Riken‐203 modulates neural development by regulating the Sox6 through sequestering miRNAs

**DOI:** 10.1111/cpr.12573

**Published:** 2019-01-22

**Authors:** Lei Zhang, Zhenyu Xue, Jia Yan, Jie Wang, Qidong Liu, Hong Jiang

**Affiliations:** ^1^ Department of Anesthesiology, Shanghai Ninth People's Hospital Shanghai JiaoTong University School of Medicine, Center for Specialty Strategy Research of Shanghai Jiao Tong University China Hospital Development Institute Shanghai China; ^2^ Anesthesia and Brain Function Research Institute Shanghai Tenth People's Hospital, Tongji University School of Medicine Shanghai China

**Keywords:** LncRNA Riken, miR‐467a‐3p, miRNA‐96, neural differentiation, Sox6

## Abstract

**Objectives:**

Long non‐coding RNAs (LncRNAs) play important roles in epigenetic regulatory function during the development processes. In this study, we found that through alternative splicing, LncRNA C130071C03Riken variants Riken‐201 (Riken‐201) and Riken‐203 (Riken‐203) are both expressed highly in brain, and increase gradually during neural differentiation. However, the function of Rik‐201 and Rik‐203 is unknown.

**Materials and methods:**

Embryonic stem cells (ESCs); RNA sequencing; gene expression of mRNAs, LncRNAs and miRNAs; over‐expression and RNA interference of genes; flow cytometry; real‐time quantity PCR; and Western blot were used in the studies. RNA pull‐down assay and PCR were employed to detect any miRNA that attached to Rik‐201 and Rik‐203. The binding of miRNA with mRNA of Sox6 was presented by the luciferase assay.

**Results:**

Repression of Rik‐201 and Rik‐203 inhibited neural differentiation from mouse embryonic stem cells. Moreover, Rik‐201 and Rik‐203 functioned as the competing endogenous RNA (ceRNA) to repress the function of miR‐96 and miR‐467a‐3p, respectively, and modulate the expression of Sox6 to further regulate neural differentiation. Knockout of the Rik‐203 and Rik‐201 induced high ratio of brain developmental retardation. Further we found that C/EBPβ might potentially activated the transcription of Rik‐201 and Rik‐203.

**Conclusions:**

These findings identify the functional role of Rik‐201 and Rik‐203 in facilitating neural differentiation and further brain development, and elucidate the underlying miRNAs‐Sox6‐associated molecular mechanisms.

## Graphical abstract

C/EBPβ might potentially activate the transcription of Rik‐201 and Rik‐203. Rik‐201 and Rik‐203 function as the competing endogenous RNA (ceRNA) to repress the function of miR‐96 and miR‐467a‐3p respectively and modulate the Sox6 expression to regulate neural differentiation.

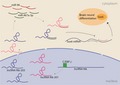

## 1 INTRODUCTION

Neural differentiation is an important developmental event during gastrulation of the embryonic development.^1^ The abnormal neural differentiation results in severe neural tube defects,^2^ which may further induce the risk of post‐natal lethality or lifelong disability.^3^ However, there are still many regulatory mechanisms should be investigated.

Embryonic stem cells (ESCs) have the capacity to generate many differentiated types of cells in the body and are excellent sources for regenerative medicine and development research. Neural stem cells (NSCs) differentiated from the ESCs could be used for treatment of Alzheimer‐s and Parkinson‐s disease.^4^, ^5^ Previous study indicated that Sirt1 regulated neural differentiation of mouse embryonic stem cells (mESCs).^3^ Sox2 mediates proliferation and neurogenesis of neural precursors derived from the ESCs by regulating Lin28.^6^ In addition to the protein‐coding genes, long non‐coding RNAs (LncRNAs) function as critical modulators in many biological processes over the course of human development by regulating complicated and multiple signalling pathways.^7^, ^8^, ^9^, ^10^ Analyses of the genome‐wide transcriptome indicate that there are thousands of LncRNAs.^11^ Many of the LncRNAs are expressed spatially and temporally, contributing to neural differentiation, further brain development or nerve‐related diseases.^8^, ^12^, ^13^, ^14^, ^15^, ^16^ However, very little is known about the specific function of LncRNAs, particularly the role of related regulating pathway in neural differentiation.

MicroRNAs (miRNAs) are 20‐25 nucleotide (nt) non‐coding RNAs that partially bind to the mRNA 3′ untranslated regions (3′UTRs) to induce the translational repression.^17^, ^18^ miRNAs are involved in many biological and physiological processes, such as the regulation of disease formation, and embryonic development.^19^, ^20^, ^21^, ^22^ miRNAs are abundant in the CNS and are critically involved in all stages of neural differentiation during brain development.^23^, ^24^ miR‐96 has been reported to repress neural induction from human embryonic stem cells (hESCs) by targeting Pax6, the critical regulator of neural differentiation.^25^ Whether miR‐96 regulated the neural differentiation and the regulatory mechanism remains unclear.

LncRNAs can be the competing endogenous RNA (ceRNA) to function as miRNA sponge by sequestrating the miRNAs and releasing them away from mRNA 3′UTR binding sites to further repress the function of miRNAs.^26^, ^27^ LncRNA ND regulates expression of Notch genes by sequestering miR‐143‐3p.^8^ LncRNAN2 was found to harbour neurogenesis‐associated miRNAs, miR‐125b and let‐7, in its intronic regions.^28^ A recent study identified the novel Rik‐201 and its homologous gene ECONEXIN,^29^ and demonstrated its ceRNA functional role in gliomagenesis. There is, however, still much that needs to be understood about the numerous mechanisms of LncRNAs in regulating neural development by functioning as the ceRNA, and inducing further influence of the downstream signalling pathways.

SOX6 is a critical neural differentiation‐related gene.It was found to regulate the specification of dopamine neurons^30^ and also controls dorsal progenitor identity and inter‐neuron diversity during neocortical development.^31^ Disruption of Sox6 is associated with dopa‐responsive movement disorder.^32^ Whether Sox6 could be regulated by miRNAs during the neural differentiation remains unknown.

## 2 MATERIALS AND METHODS

### 2.1 Establishment of inducible LncRNA interference knockdown mESCs lines

For downregulation of Rik‐201:ENSMUST00000131907.8, Rik‐203:ENSMUST00000182788.1, NR_015561.2, we designed the specific 21 nucleotides targeting the transcripts of LncRNA Rik‐201(shRNA1,5′‐GCACACACTAAGACAGAAACC‐3′; shRNA2, 5′‐GCTGTCACTTCTATAGAATGG‐3′); LncRNA Rik‐203 (shRNA‐1, 5′‐GGTGTTGGGCCAGTTCCTTAT‐3′; shRNA‐2, 5′‐GCTTGAATTCAGGCTGCTTGA‐3′). Genomeditech (China) synthesized the oligos of shRNA and cloned each of them into the pLKO‐Tet‐On. Using the methods described in our previous study,^33^, ^34^ we generated the mESCs lines. Specifically, the 46C mESCs^33^ were cultured in the knockout‐DMEM (Gibco, New York, NY, USA) medium with 15% foetal bovine serum (FBS; Gibco), 1% non‐essential amino acids (NEAA; Thermo Fischer Scientific, Waltham, MA, USA), 1% L‐glutamine (Thermo Fischer Scientific), 1% sodium pyruvate (Thermo Fischer Scientific), 55 μmol/L β‐mercaptoethanol (Gibco) and leukaemia inhibitory factor (LIF) (Millipore) at 37°C, 5% CO_2_ atmosphere. The mESCs were dissociated with 0.05% trypsin and transfected at a density of 5000/mL with rtTA lentivirus (10^6^ transducing units/mL) supplemented with 8 μg/mL polybrene (Qcbio Science & Technologies, Shanghai, China). Forty‐eight hours later, cells were selected using geneticin (G418 Sulfate, 50 mg/mL; Thermo Fischer Scientific). A stably transfected cell line was selected and infected with pLKO‐Tet‐On‐lncRNA shRNA lenti‐virus or pLKO‐Tet‐On empty lentivirus for 48 hours with 8 μg/mL polybrene before selection with 5 μg/mL puromycin (Sigma‐Aldrich, Shanghai, China). The medium was changed every day for 7 days until the single‐cell clone could be identified under a microscope. Clones were picked up and dissociated with trypsin and plated onto feeder cell‐coated 24‐well plates.

### 2.2 Neural differentiation form mESCs

We performed the neural differentiation of mESCs by using the methods described in our previous study.^33^, ^34^ Specifically, 46C mESCs were dissociated into single cells using 0.05% trypsin (Gibco) and then neutralized with DMEM (Gibco) containing 10% FBS. After being counted, mESCs were washed with GMEM (Gibco, USA) and re‐suspended in a Petri dish at a density of 25 000‐50 000/mL using the neural differentiation medium GMEM with 8% Knockout Serum Replacement (KOSR; Gibco), 1% L‐glutamine, 1% sodium pyruvate, and 0.1 mmol/L β‐mercaptoethanol. The medium was changed every 2 days. We added the Dox for downregulating the lncRNA begin at day 3 during the neural differentiation.

### 2.3 Sevoflurane anaesthesia for mice

We used C57BL/J6 mice at post‐natal day 6 (Shanghai SLAC Laboratory Animal, Shanghai, China) for studies. The protocol was according to Standing Committee on Animals at Shanghai Ninth People‐s Hospital, Shanghai, China. Mice received sevoflurane anaesthesia as our previous studies. The brain tissues of mice were harvested after sevoflurane anaesthesia with 4.1% sevoflurane for 2 hours at day 4, 5 and 6 after the start of neural differentiation.^35^

### 2.4 Flow cytometry studies

The cells were suspended in PBS for flow cytometry analysis by using FACS Calibur (BD Biosciences, Franklin Lakes, NJ, USA) operating at 488 nm excitation with standard emission filters. Fluorescence noise baseline was referenced with the 46CmESCs. FlowJo software (Stanford, CA, USA) was used to analyse the results.

### 2.5 Reverse transcription PCR and real‐time quantity PCR

RNA was extracted using RNAiso Plus (TaKaRa, Dalian, China). Inverse transcription of mRNA to cDNA was performed by using a cDNA Synthesis Kit (TaKaRa). Inverse transcription of miRNAs to cDNA was carried out through the TIANScript RT Kit (Tiangen, Beijing, China). The PCR primers of miRNA were purchased (RiboBio, Guangzhou, China). Primers for the qRT‐PCR analysis of mRNA are as follows:

**Table:** 

Rik‐201	Forward	5′‐GAACAGACTATTCAAACAAAGGTAA‐3′
	Reverse	5′‐GTGAGAGATTGTAGACCTGTTTTCC‐3′
Rik‐203	Forward	5′‐CATCACTTGGACCATGGACACTAAT‐3′
	Reverse	5′‐GAATCCTATACACATGAATGCAGAA‐3′
Sox1	Forward	5′‐GTTTTTTGTAGTTGTTACCGC‐3′
	Reverse	5′‐GCATTTACAAGAAATAATAC‐3′
Nestin	Forward	5′‐GAATGTAGAGGCAGAGAAAACT‐3′
	Reverse	5′‐TCTTCAAATCTTAGTGGCTCC‐3′
GAPDH	Forward	5′‐ATGACATCAAGAAGGTGGTG‐3′
	Reverse	5′‐CATACCAGGAAATGAGCTTG‐3′

### 2.6 Nuclear and cytoplasm extraction

We performed the nuclear and cytoplasm extraction studies using the methods described in our previous study.^36^ Specifically, 1 × 10^8^ mESc‐derived NPCs were prepared for this assay. The cells were washed three times with the phosphate buffered saline. Then, working reagents buffer I and buffer II, respectively, were added to the pellets and mixed gently. The tube containing the cells was placed into an freezer and shaken at 200 rpm on a platform for 2 hours. The samples were centrifuged at 12 000 × *g* for 5 minutes at 48°C, and finally, the nuclear and cytoplasm extract was obtained. Then, purification and analysis of cytoplasmic and nuclear RNA was performed using quantitative RT‐PCR.

### 2.7 RNA pull‐down assay

1 × 10^8^ mESc‐derived NPCs were used for the study. Full‐length C130071C03Rik and its antisense RNA were transcribed into the cells using T7 RNA polymerase. 50 pmol of C130071C03Rik, and C130071C03Rik‐s antisense RNA, was labelled utilizing desthiobiotin and T4 RNA ligase using Pierce RNA 3′End Desthiobiotinylation Kit (Thermo Fischer Scientific). The RNA pull‐down assay was performed according to the Pierce Magnetic RNA‐Protein Pull‐Down Kit (Thermo Fischer Scientific). In addition, the cells were briefly lysed with Pierce IP Lysis Buffer, and incubated on ice for 5 minutes. The lysates were centrifuged at 13 000 × *g* for 10 minutes, and the supernatant was transferred to a new tube for further analysis. The labelled RNA was added to 50 μL of beads, and incubated for 30 minutes at room temperature with agitation. The RNA‐bound beads were incubated with the lysates for 60 minutes at 4°C. The RNA‐Binding microRNAs were washed and eluted, and the binding microRNAs were detected using qRT‐PCR. Primers for the qRT‐PCR analysis of miRNA include the following list. For miR‐96:

Primer of Stem‐loop reverse transcription: 5′‐GTCGTATCCAGTGCGTGTCGTGGAGTCGGCAATTGCACTGGATACGACAGCAAAA3′, Primer of qRT‐PCR: PF: 5′‐CGCAGTTTGGCACTAGCACAT‐3′, RF: 5′‐AGTGCGTGTCGTGGAGTCG‐3′. For miR‐467a‐3p: Primer of Stem‐loop reverse transcription: 5′‐GTCGTATCCAGTGCGTGTCGTGGAGTCGGCAATTGCACTGGATACGACTGTAGGT‐3′, Primer of qRT‐PCR: PF: 5′‐CGGCGGCATATACATACACACA‐3′, RF: 5′‐AGTGCGTGTCGTGGAGTCG‐3′.

### 2.8 Luciferase reporter assays

For lncRNA‐miRNA binding site luciferase reporter construction:

Fragments of the 3′UTR of lncRNA Rik‐203 were amplified from the DNA of NPCs by PCR, with the primers as follows: PF: 5′‐GGCGAGCTCGAGATTACTTGCTGGAAGGGGA‐3′, with a sacI restriction site; reverse: 5′‐GGCCTCGAGCGTGGGAATCGGAGCGTC‐3′ with an xbaI restriction site. Fragments of the 3′UTR of lncRNA Rik‐201 were also amplified from the DNA of NPCs by PCR, with the primers as follows: PF: 5′‐GGCGAGCTCAGAAGCTCCTATTTAGAGGAAAGGG‐3′; PR: 5′‐GGCCTCGAGGGATATACTGAATTCAAGCAGCCTG‐3′. The fragments were inserted into the pGL3‐cm vector (Promega, Madison, WI, USA). The mutant binding site sequence luciferase reporter was generated by replacing the miRNAs binding site sequence with miRNAs seed sequences and insertion of the mutant sequence into luciferase reporter vector pGL3cm.

For mRNA‐miRNA binding site luciferase reporter construction:

pGl3‐cm vector was also used to construct the mRNA 3′UTR luciferase reporter. The fragment of Sox6 3′UTR was amplified from the DNA of mESCs by the primers in the following list. For miR‐96 binding sites UTR region: PF: 5′‐GGCGTCGACGATTTCGTATTGTGAAACCGG‐3′, PR: 5′‐GGCTCTAGA TTTGCTGTTTTATTTTAAGATGTCA‐3′. For miR‐467a‐3p binding sites UTR region: PF: 5′‐GGCGTCGACCCCTCCAGTGGGACTTGTCC‐3′, PR: 5′‐GGCTCTAGACACTCCATCTTTTGTACTGCTGTTG‐3′. The mutant UTR reporter vector was obtained by replacing the miRNA‐binding site sequences using the Quik Change Site‐Directed Mutagenesis Kit (Agilent Stratagene, Santa Clara, CA, USA). 3T3 cells (5 × 10^4^ cells per well in 24 well plates) were transfected with 350 ng of the luciferase reporter, 5 ng Renilla vector, and 50 pmol of miR‐96 or miR‐467a‐3p mimics or control miRNA mimics (Biotend, Shanghai, China) using Lipofectamine 2000 (Thermo Fischer Scientific). Twenty‐four hours after the co‐transfection, the cells were harvested, and the luciferase activity was analysed using the Dual Luciferase Assay Kit (Promega). The luciferase activity was detected by a Spectra Max M5 microplate reader (Molecular Devices, San Jose, CA, USA).

### 2.9 Western blotting

Cells were lysed using an SDS buffer (Beyotime, Shanghai, China) to obtain the protein for electrophoresis. The whole protein was then transferred onto the PVDF membrane (Whatman, Kent, England). Primary antibodies that were used in incubation include AnGAPDH (ab8245; Abcam, Cambridge, England) antibody, which was used for normalizing the protein levels, and Sox6 antibody (14010‐1‐AP; Proteintech, Rosemount, IL, USA). Protein expression signalling was visualized through enhanced chemiluminescence (ECL) substrate (Thermo Fischer Scientific).

### 2.10 Over‐expression of Sox6 by pcDNA3.1‐Sox6 vector

The whole RNA was isolated, and inverse transcription to cDNA was then performed using cDNA Synthesis Kit (TaKaRa). Sox6 CDS fragments were amplified and inserted into the pcDNA3.1 vector. The primers sequence includes the following list: PF: 5′‐GGCGCTAGCATGTCTTCCAAGCAAGCCACCT‐3′ (restriction enzyme site, Nhe1), PR: 5′‐GGCCGGCCGTCAGTTGGCACTGACAGGCTC‐3′ (restriction enzyme site, HindIII). The vector was transfected into the cells using Lipofectamine 2000 (Thermo Fischer Scientific), and the instructions for the reagent.

### 2.11 Over‐expression of miR‐96 and miR‐467a‐3p

We transfected the miR‐96 and miR‐467a‐3p mimics overexpressed vector (Biogot Technology, Nanjing, China) into the embryonic bodies derived from 46c mESCs during the neural differentiate on every 48 hours to maintain the certain concentration using Lipofectamine 2000 (Thermo Fischer Scientific), following the instructions given to overexpress the miR‐96 and miR‐467a‐3p. The expression level of over‐expression is detected by qRT‐PCR.

### 2.12 Sevoflurane anaesthesia for mice

The protocol was approved by the Standing Committee Shanghai of The Ninth People‐s Hospital (Shanghai, China) on the Use of Animals in Research and Teaching. C57BL/J6 mice at post‐natal day 6 (P6) (Shanghai Laboratory Animal Center, Zhangjiang, Shanghai, China) were used in the studies. The mice received the Sevoflurane anaesthesia as described in our previous studies.^35^, ^37^ The brain tissues of mice were harvested at the end of the Sevoflurane anaesthesia administration.

### 2.13 Inhibition of miR‐96 and miR‐467a‐3p

In order to inhibit the miRNA function, we transfected the miRNA‐96 or miR‐467a‐3p inhibitor (Ribobio, Guangzhou, China) by Lipofectamine 2000 (Thermo Fischer Scientific) into the cells during the neural differentiation every 48 hours. miRNA inhibitor is the synthetic double‐stranded nucleic acid which can directly bind with the target miRNA by complementary base pairing to compete with the miRNA downstream target mRNA, promoting the mRNA translation.

### 2.14 Production of the Rik‐201 and Rik‐203 knockout mice

LncRNA Rik‐201 and Rik‐203 knockout mice by using CRISPR/Cas9 system were purchased from CasGene Biotech, Ltd. Beijing, China. sgRNA was designed for targeting the front sequence of second and the end of the third exon of the C130071C03Rik and inserted into pX330 vector. The T7 promoter‐derived sgRNA DNA fragments were amplified for further in vitro transcription by using MEGA short scriptTM T7 Transcription Kit (Invitrogen AM1354, Carlsbad, CA, USA). 50 ng sgRNA and 100 ng Cas9 RNAs were injected into the isolated zygotes which were then implanted into pseudopregnant C57BL/6 mice. The sequence of gRNA were as follows: 5′‐CAATAAAAGGCGATCGCTCCAGG‐3′, 5′‐TAACCGAGATGCGACCTTCGTGG‐3′. The PCR primers for verifying the offspring genome DNA editing are as follows: PF: 5′‐GCCCACAGAACAGGAGCCGAAACAAC‐3′; PR 5′‐CCTGACCCCTCCAGACAGTATTCTATTCAAACA‐3′.

### 2.15 Statistics

The data were presented as mean + standard deviation (SD) with more than three independent experiments. The significance of statistics was determined by a student‐s *t* test, one‐way ANOVA and two‐way ANOVA. * and #*P* < 0.05, **and ##*P < *0.01, *** and ###*P* < 0.001. The studies employed two‐tailed hypothesis, and statistically significant *P* values were <0.05. We used GraphPad (GraphPad Software, La Jolla, CA, USA) to evaluate all of the study data.

## 3 RESULTS

### 3.1 LncRNA Rik‐201 and Rik‐203 is critically involved in mouse neural differentiation

**Figure 1:**
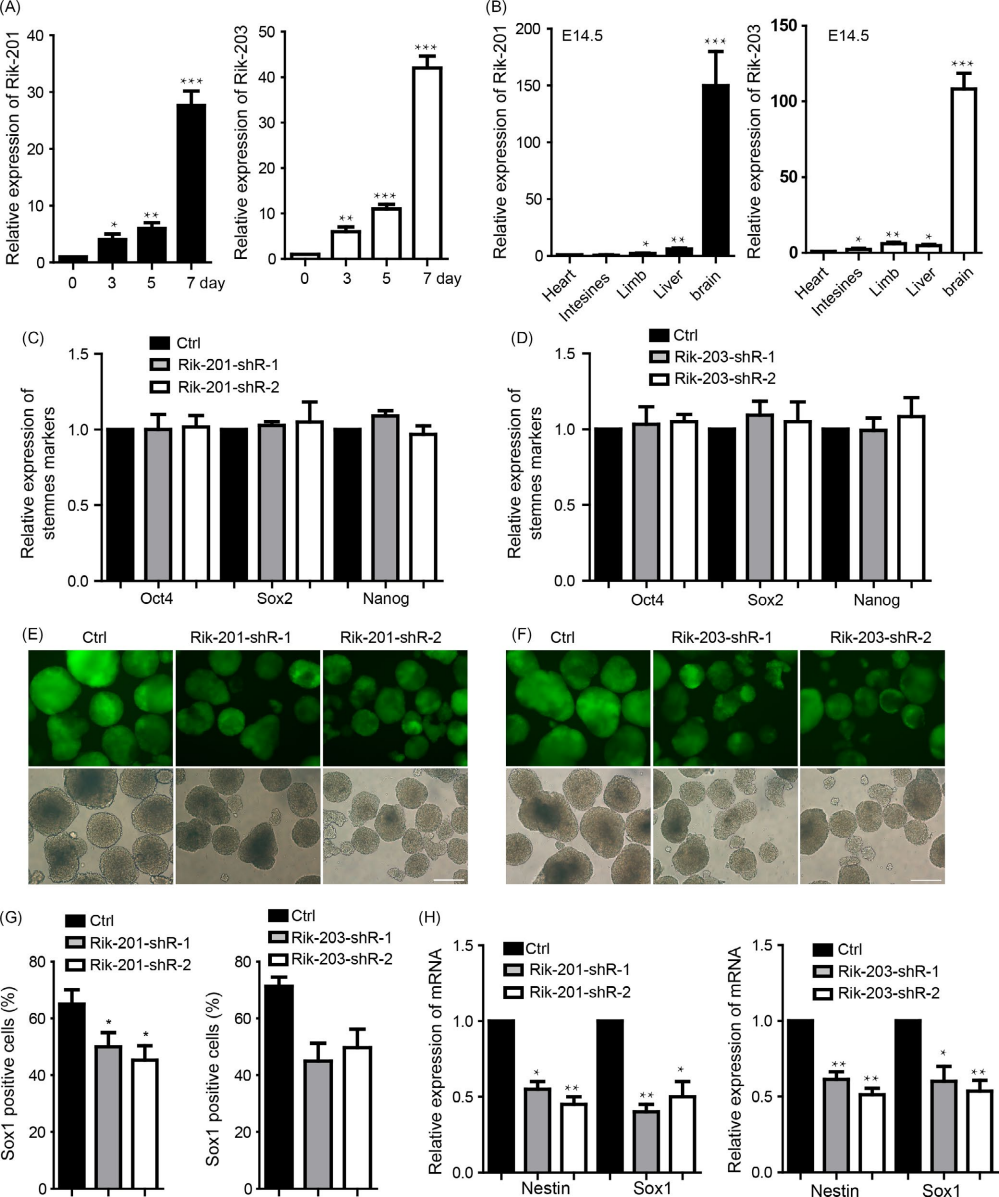
LncRNA Riken is critically involved in the mouse neural differentiation. A, The increase of Rik‐201 and Rik‐203 expression compared with the mESCs (day 0 cells) during the neural differentiation from ESCs to NPCs. B, Rik‐201 and Rik‐203 levels were detected by RT‐PCR in different tissues of mice; the brain has the highest levels of Rik‐201 and Rik‐203 compared with the level in heart. C, The Rik‐201 and (D) Rik‐203 knockdown mESCs lines both showed that there was no significant change in the mESCs self‐renewal. ctrl means the empty plko‐Tet‐On vector control group. E, Representative pictures showed that Rik‐201 and (F) Rik‐203 knockdown inhibited the neural differentiation. G, Measurement of Sox1‐positive cells indicated that knockdown of Rik‐201 and Rik‐203 decreased the number of Sox1‐positive cells, which was compared with the empty vector control group. H, RT‐PCR showed that the mRNA levels of Sox1 and Nestin were decreased through knockdown of Rik‐201 and Rik‐203. The scale bar represents 100 μm. Ctrl, control; ESCs, embryonic stem cells; FACS, fluorescent‐activated cell sorting; GFP, green fluorescent protein; NPCs, neural precursor cells; Rik‐201, C130071C03Riken‐201; Rik‐203, C130071C03Riken‐203; shRNA, short hairpin RNA; Sox1, SRY (sex determining region Y)‐box 1. Data are represented as mean ± SEM. *P < 0.05, **P < 0.01, ***P < 0.001; by one‐way ANOVA (A and B), by *t* test (C, D, G and H). For A and B, n = 5 independent experiments. For C, D, G and H, n = 3 independent experiments

We performed neural differentiation of the mESCs to neural progenitor cells (NPCs), and found that alternative splicing of LnRNA‐Rik (C130071C03Riken) variants, Rik‐201 and Rik‐203, was up‐regulated during neural differentiation from day 3 (Figure 1A). Additionally, these two variants were also more enriched in the embryonic brain than in other tissues at day 14.5 (Figure 1B). We constructed the Rik‐201 and Rik‐203 knockdown mESCs lines, respectively, and found that there was no significant change in the stemness marker expression of the mESCs compared with the control mESC with only empty pLKO‐tet‐on vector (Figure 1C,D). We performed the neural differentiation derived from the mESCs lines and found that downregulation of Rik‐201 and Rik‐203 significantly inhibited the neural differentiation derived from these mESC lines (Figure 1E,F). FACS studies further indicated the reduced amounts ratio of Sox1‐positive cells detected at day 7 during the neural differentiation (Figure 1G).The NPCs related markers Sox1 and Nestin detected at day 7 were also downregulated (Figure 1H).

### 3.2 miR‐96 and miR‐467a‐3p combine with Rik‐201 and Rik‐203 respectively and both repress neural differentiation

**Figure 2:**
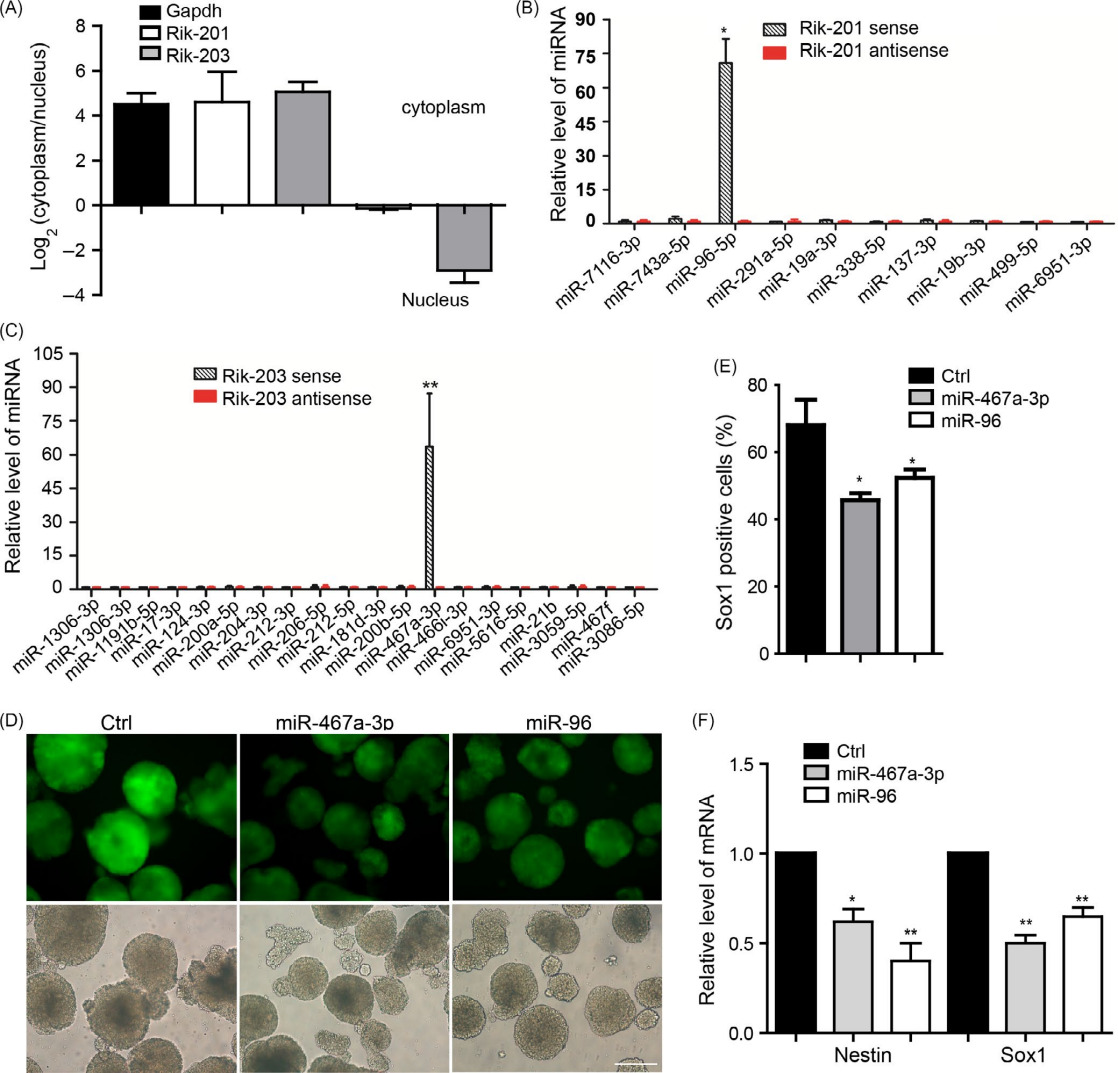
miR‐96 and miR‐467a‐3p combine with Rik201 and Rik‐203, respectively, and both repress the neural differentiation. A, Detection of cytoplasmic and nuclear distribution of Rik‐201 and Rik‐203 by fractionation. RT‐PCR showed that most of Rik‐201 and part of Rik‐203 located in the cytoplasm. B, RNA pull‐down experiments determined that miR‐96 combined with Rik‐201. C, miR‐467a‐3p highly combined with the Rik‐203. D, Measurement of Sox1‐positive cells indicated that over‐expression of miR‐96 or miR‐467a‐3p repressed the neural differentiation and decreased the number of Sox1‐positive cells. E, The quantification of Sox1‐positive cells using FACS showed over‐expression of miR‐96 or miR‐467a‐3p decreased the number of Sox1‐positive cells. F, RT‐PCR showed that the mRNA levels of Sox1 and Nestin were decreased through over‐expression of miR‐96 or miR‐467a‐3p. The scale bar represents 100 μm. Ctrl, control; Data are represented as mean ± SEM *P < 0.05, **P < 0.01, ***P < 0.001; by one‐way ANOVA (E and F). n = 3 independent experiments

A recent study identified the novel Rik‐201 and its homologous gene ECONEXIN and demonstrated its ceRNA functional role in gliomagenesis.^29^ We also found that most of Rik‐201, and part of Rik‐203, are located in the cytoplasm (Figure 2A), which suggest they might function as the ceRNA. RNA pull‐down experiments determined that miR‐96 combined with Rik‐201 (Figure 2B), and miR‐467a‐3p combined with Rik‐203 (Figure 2C). Over‐expression of miR‐96 and miR‐467a‐3p via transfecting the synthetic miRNA mimics (Figure S1A,B) repressed the neural differentiation derived from mESCs (Figure 2D). We also performed the luciferase reporter assay to further confirm the interaction of miRNAs and lncRNA. The results indicated that miR‐476a‐3p and miR‐96 could bind to the Rik‐203 and Rik‐201, respectively (Figure S1C). FACS studies further indicated that miR‐96 or miR‐467a‐3p reduced amounts of Sox1‐positive cells (Figure 2E). The expression of NPCs markers Sox1 and Nestin was also downregulated (Figure 2F). We further constructed the miRNA sponge vectors that contain complementary binding sites to a miRNA^38^, ^39^ to create continuous miR‐467a‐3p or miR‐96 loss of function. Then, we made the stable miRNA sponge ESCs line to perform the further neural differentiation and found that the inhibition of miR‐467a‐3p or miR‐96 promotes the efficiency of neural differentiation compared with the empty vector group (Figure S1D).

### 3.3 miR‐96 and miR‐467a‐3p mediated the function of Rik201 and Rik‐203 respectively in regulating neural differentiation

**Figure 3:**
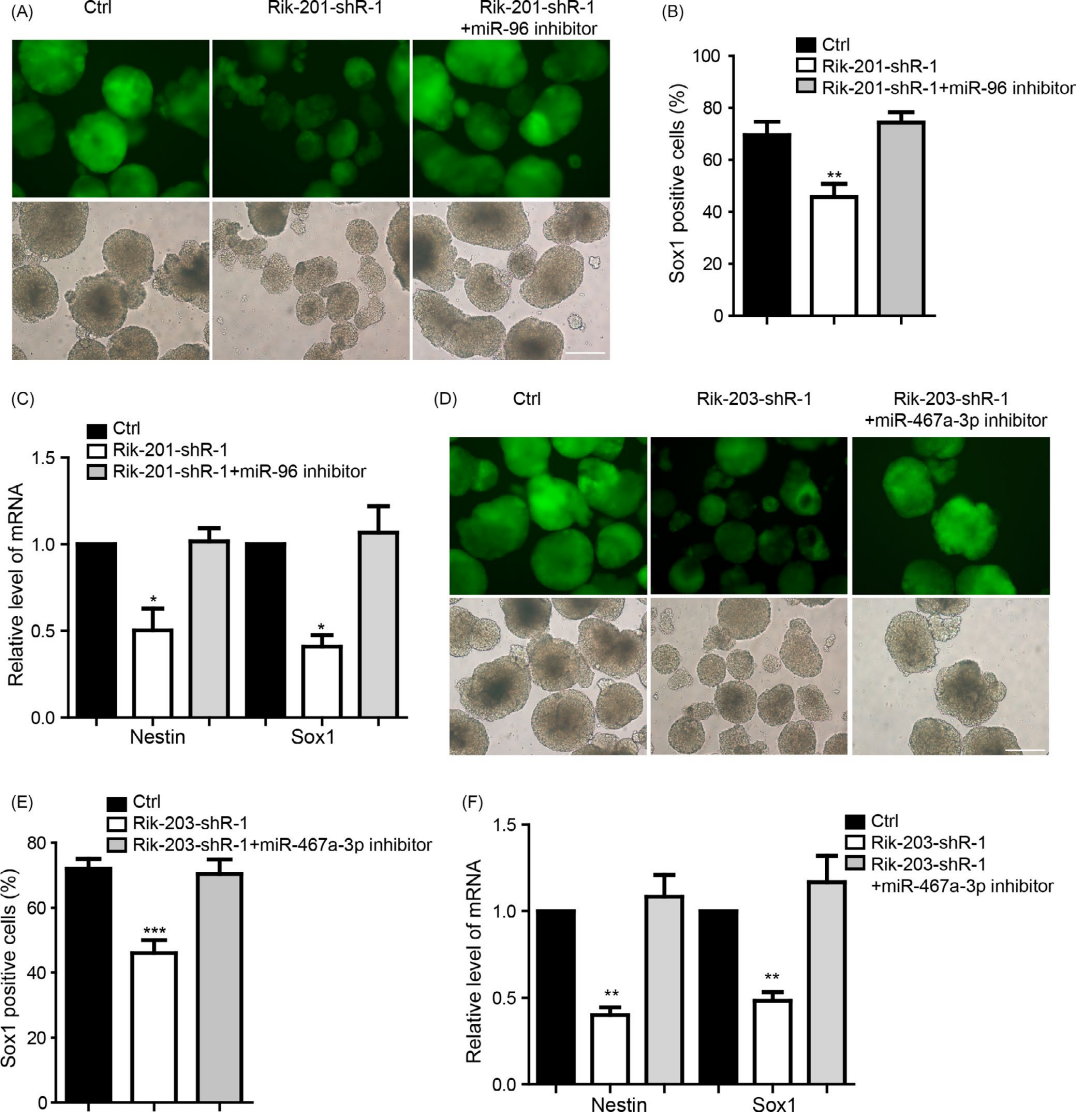
miR‐96 or miR‐467a‐3p mediated the function of Rik201 and Rik‐203, respectively, in regulating the neural differentiation. A, The miR‐96 inhibitor significantly restored the neural differentiation repression, and the Sox1‐positive cells decrease was caused by Rik‐201. Ctrl means the cells with empty plko‐Tet‐On vector and were transfected with miRNA inhibitor control and B, The quantification of Sox1‐positive cells using FACS indicated that knockdown Rik‐201 reduced Sox1‐positive cells, and that miRNA‐96 inhibitor prevented such reductions. C, RT‐PCR showed that the mRNA levels of Sox1 and Nestin repressed by Rik‐201 knockdown were also rescued by miR‐96 inhibitor. D, The miR‐467a‐3p inhibitor significantly restored the neural differentiation repression and the Sox1‐positive cells decrease caused by Rik‐203. E, The quantification of Sox1‐positive cells using FACS indicated that knockdown Rik‐203 reduced Sox1‐positive cells, and that miR‐467a‐3p inhibitor mitigated such reductions. F, RT‐PCR showed that the mRNA levels of Sox1 and Nestin repressed by Rik‐203 knockdown were also rescued by miR‐467a‐3p inhibitor. The scale bar represents 100 μm; Data are represented as mean ± SEM.*P < 0.05, **P < 0.01, ***P < 0.001; by one‐way ANOVA (B, C, E and F). n = 3 independent experiments

We further performed the rescue experiments to detect whether inhibition of miR‐96 and miR‐467a‐3p mediated the function of knockdown of Rik‐201 and Rik‐203, respectively. We transiently transfect the miR‐96 inhibitor during the neural differentiation at day 7 to detect the function of miR‐96 inhibition via detecting the upregulation of EGR‐1 expression (Figure S2A) which has reported in previous study.^40^ Then, we performed the neural differentiation from the Rik‐201 knockdown mESCs lines and co‐transfected the miR‐96 inhibitor or control inhibitor for every 48 hours during the process of neural differentiation. Result indicated that inhibition of miR‐96 could significantly restore the neural differentiation repression caused by Rik‐201 knockdown, which was detected at day 7 (Figure 3A). FACS studies also indicated that knockdown of Rik‐201 reduced Sox1‐positive cells, and that miRNA‐96 inhibitor mitigated such reductions (Figure 3B). The expression of NSCs markers Sox1 and Nestin was reduced by Rik‐201 knockdown, which was also rescued by inhibition of miR‐96 (Figure 3C). Further, we detected the miR‐467a‐3p inhibitor effect in mouse NIH3T3 cells via detecting the upregulation of Fas reported to be the miR‐467a target in previous study^42^ (Figure S2B). Inhibition of miR‐467a‐3p restored the neural differentiation that was repressed by Rik‐203 knockdown (Figure 3D). FACS studies further indicated that knockdown of Rik‐203 reduced the rate of Sox1‐positive cells, and that miRNA‐467a‐3p inhibitor rescued such reductions (Figure 3E) and also the marker expression of the NPCs (Figure 3F).

### 3.4 miR‐96 and miR‐467a‐3p target the Sox6 to control over the neural differentiation

**Figure 4:**
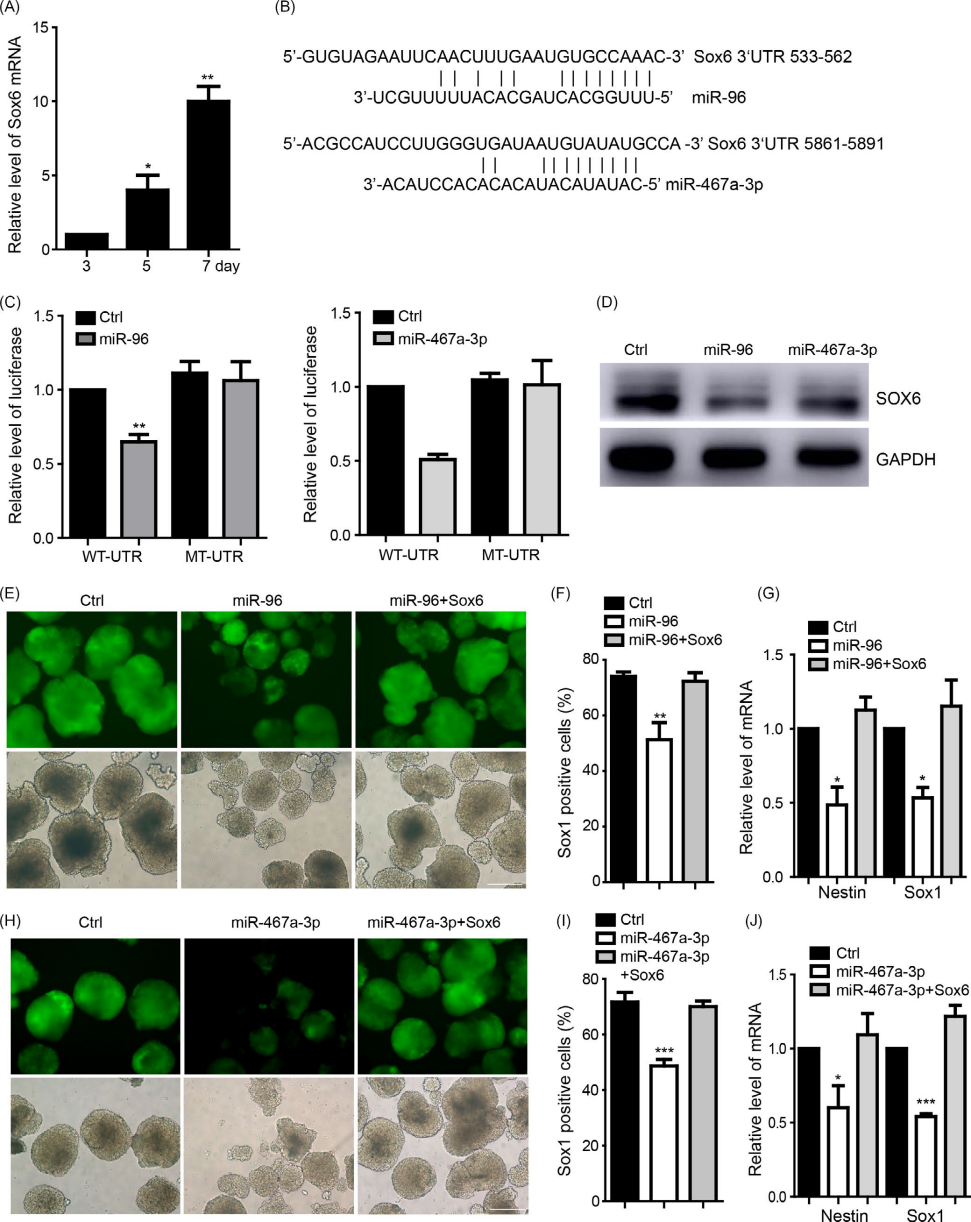
miR‐96 and miR‐467a‐3p target the Sox6 to regulate the neural differentiation. A, The expression of Sox6 during the neural differentiation from ESCs to NPCs. B, Target validation of the binding of Sox6 3′UTR by miR‐96 or miR‐467a‐3p. C, Luciferase report assay indicated that miR‐96 or miR‐467a‐3p targeted wild‐type Sox6 3′UTR but not mutant UTR. D, Over‐expression of miR‐96 or miR‐467a‐3p decreased the protein level of Sox6. E, The Sox6 restored the neural differentiation repression and the Sox1‐positive cells decrease caused by over‐expression of miR‐96. F, The quantification of Sox1‐positive cells using FACS indicated that over‐expression of miR‐96 reduced Sox1‐positive cells, and that Sox6 mitigated such reductions. G, RT‐PCR showed that the mRNA levels of Sox1 and Nestin repressed by over‐expression of miR‐96 were also rescued by Sox6. H, The Sox6 restored the neural differentiation repression and the Sox1‐positive cells decrease caused by over‐expression of miR‐467a‐3p. I, The quantification of Sox1‐positive cells using FACS indicated that over‐expression of miR‐467a‐3p reduced Sox1‐positive cells, and that Sox6 mitigated such reductions. J, RT‐PCR showed that the mRNA levels of Sox1 and Nestin repressed by over‐expression of miR‐467a‐3p were also rescued by Sox6. The scale bar represents 100 μm. Ctrl, control; Data are represented as mean ± SEM. *P < 0.05, **P < 0.01, ***P < 0.001; by one‐way ANOVA (A, C, F, G, I and J). n = 3 independent experiments

We detected the potential downstream targets of the miR‐96 and miR‐467a‐3p by using online miRNA target predication tools (miRanda, targetscan, miRBD) and found that miR‐96 and miR‐467a‐3p could both target Sox6 3′UTR (Figure 4A). During the neural differentiation, Sox6 was up‐regulated (Figure 4B). Luciferase reporter assay also showed that miR‐96 or miR‐467a‐3p binds to the wild‐type 3′UTR of Sox6 to downregulate the luciferase expression, but has no influence on the mutant one (Figure 4C). We further confirmed that over‐expression of miR‐96 and miR‐467a‐3p downregulated the Sox6 protein expression in the NPCs (Figure 4D). We then performed the rescue experiment to confirm that over‐expression of Sox6 restored miR‐96 inducing downregulation level of protein (Figure S3A) and also blocked the neural differentiation inhibition caused by miR‐96, which was detected at day 7 (Figure 4E,F). The decrease of the related NPCs markers influenced by miR‐96 was also rescued (Figure 4G). Additionally, over‐expression of Sox6 blocked miR‐467a‐3p inducing downregulation level of protein (Figure S3B) to restore the neural differentiation inhibited by miR‐467a‐3p (Figure 4H,I) and also the regulation of NPCs markers (Figure 4J).

### 3.5 Sox6 restored the neural differentiation repressed by downregulating Rik‐201 or Rik‐203

**Figure 5:**
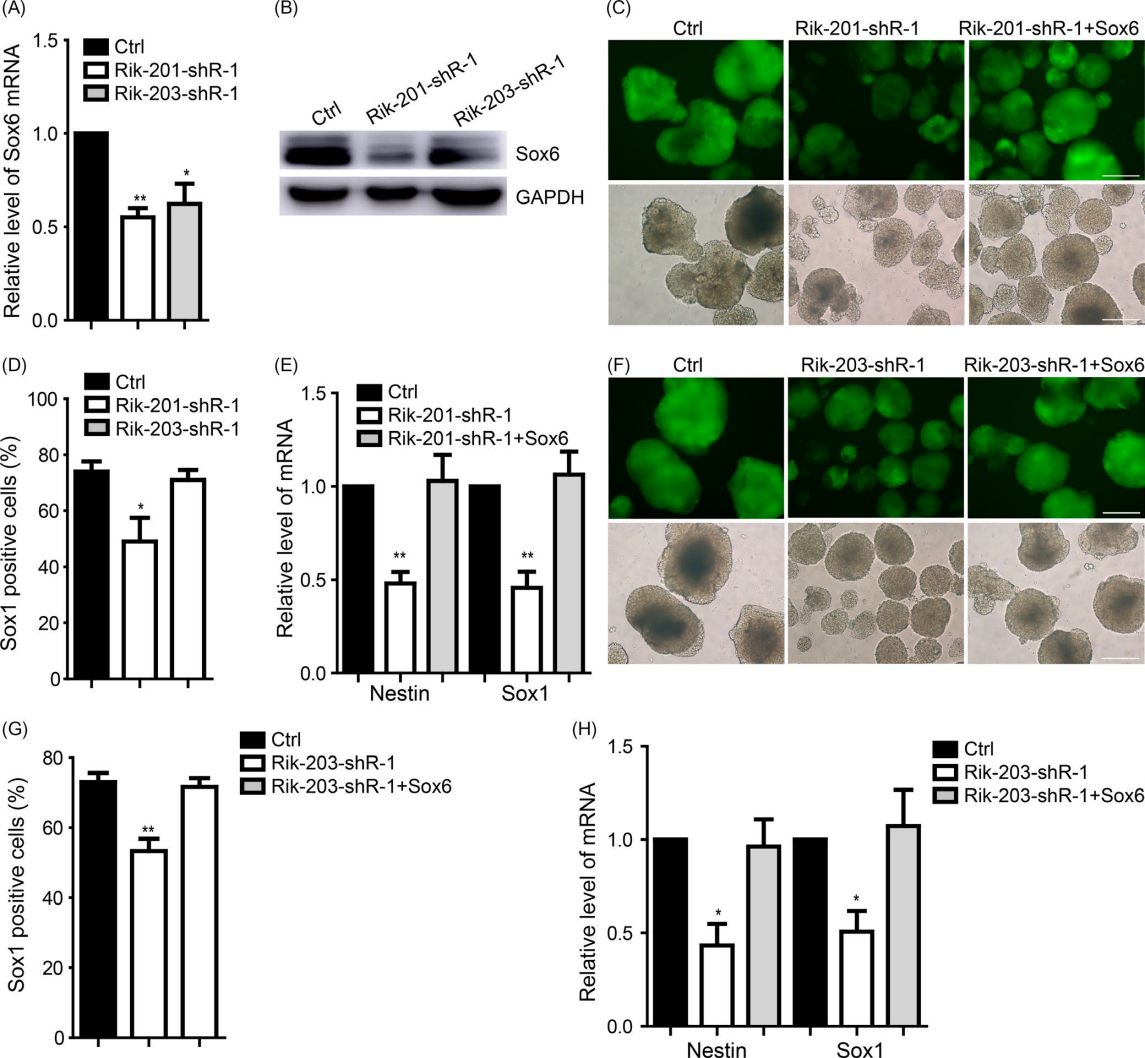
Sox6 restored the neural differentiation repressed by downregulating Rik‐201 or Rik‐203. A, Knockdown of Rik‐201 or Rik‐203 inhibited the Sox6 mRNA and (B) protein level during the neural differentiation. C, The Sox6 restored the neural differentiation repression and the Sox1‐positive cells decrease caused by knockdown of Rik‐201. D, The quantification of Sox1‐positive cells using FACS indicated that knockdown of Rik‐201 reduced Sox1‐positive cells. E, RT‐PCR showed that the mRNA levels of Sox1 and Nestin repressed by knockdown of Rik‐201 were also rescued by Sox6. F, The Sox6 restored the neural differentiation repression and the Sox1‐positive cells decrease caused by knockdown of Rik‐203. G, The quantification of Sox1‐positive cells using FACS indicated that knockdown of Rik‐203 reduced Sox1‐positive cells, and that Sox6 mitigated such reductions. H, RT‐PCR showed that the mRNA levels of Sox1 and Nestin repressed by knockdown of Rik‐203 were also rescued by Sox6. The scale bar represents 100 μm. Ctrl, control; Sox1: Data are represented as mean ± SEM. *P < 0.05, **P < 0.01, ***P < 0.001; by *t* test (A); by one‐way ANOVA (C, D, F and G). n = 3 independent experiments

A knockdown of Rik‐201 or Rik‐203 inhibited the Sox6 expression during neural differentiation, which is detected at day 7 (Figure 5A,B). Over‐expression of Sox6 also restored the inhibition of process of neural differentiation (Figure 5C), reduction of Sox1‐positive cells amounts ratio (Figure 5D), and related NPC markers levels (Figure 5E) caused by Rik‐201 knockdown. Over‐expression of Sox6 also rescued the Rik‐203 knockdown induced inhibition of process of neural differentiation (Figure 5F). Sox6 also rescued the reduction of Sox1‐positive cells (Figure 5G) and related NPCs markers level (Figure 5H) caused by knockdown of Rik‐203.

### 3.6 Knockout of the Rik‐201 and Rik‐203 induced the abnormal brain development

**Figure 6:**
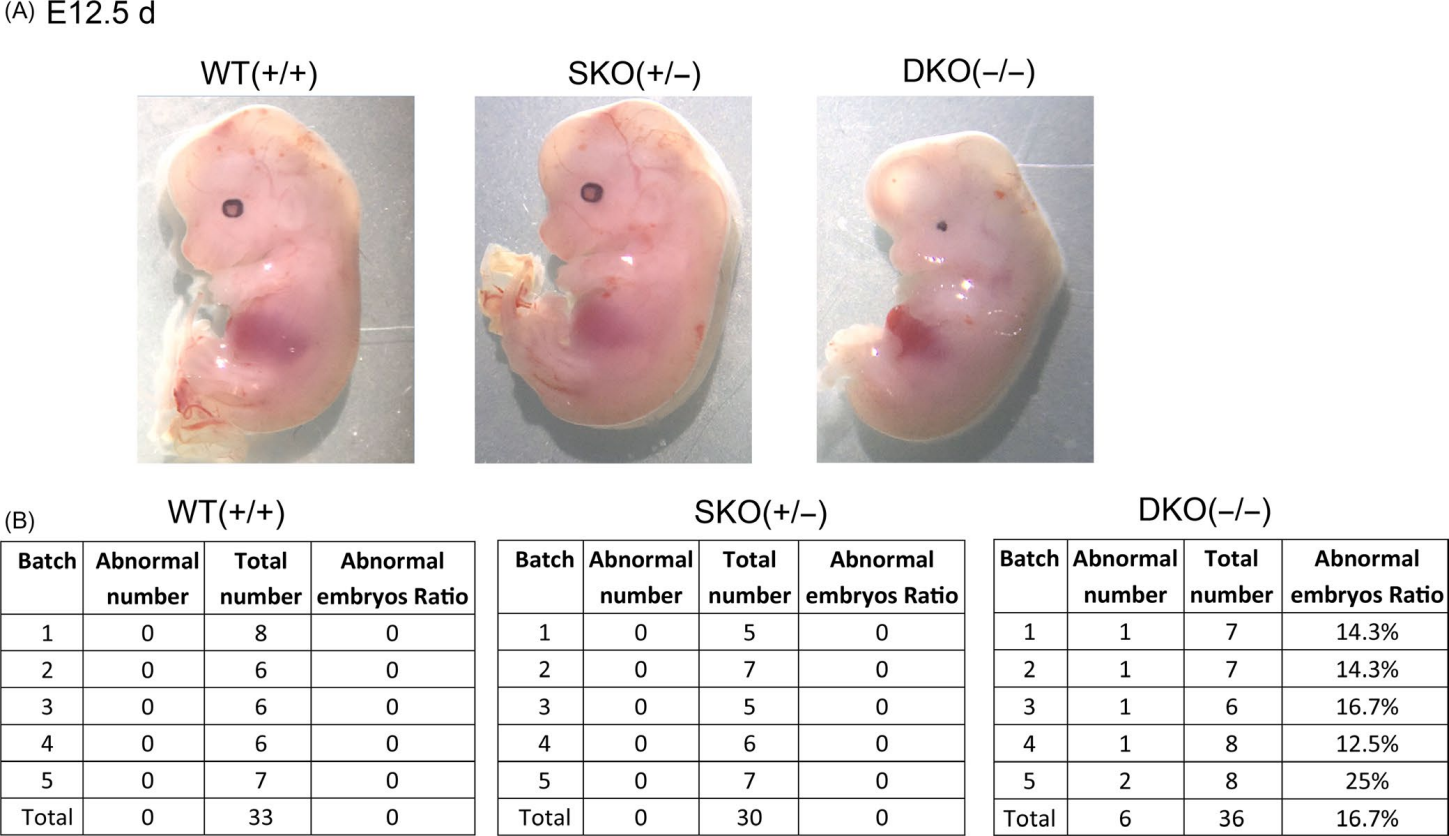
Knockout of the Rik‐201 and Rik‐203 induced the abnormal brain development. A, There is significant high ratio of brain developmental retardation in Rik‐201 and Rik‐203 knockout homozygous mice embryo at day 12.5 (E 12.5 d). There is no abnormal embryo in heterozygous and normal mouse. B, There are 16.7% of the abnormal embryos in homozygous (double knockout) mice. The heterozygote mice (single knockout) showed no abnormal development. WT (+/+) means single normal mice embryo, SKO (±) means single knockout mice embryo, and DKO (−/−) means double knockout mice embryo

The in vivo experiments of the embryonic development in Rik‐201 and Rik‐203 knockout mice showed that we constructed the Rik‐201 and Rik‐203 double knockout mice and found that there is higher ratio of embryonic brain developmental retardation in homozygous mice embryo at day 12.5. However, there is no abnormal embryo in wild‐type and heterozygous mice (single knockout; Figure 6A). Statistics indicated that there are 16.7% of the abnormal embryos during the embryonic development of homozygous mice (Figure 6B).

### 3.7 Rik‐201 and Rik‐203 may be regulated by C/EBPβ and associated with anaesthesia‐induced neurotoxicity

**Figure 7:**
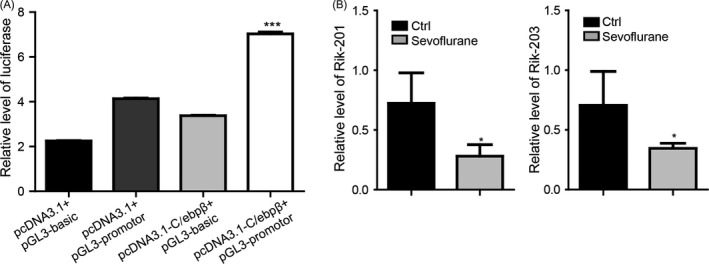
Rik‐201 and Rik‐203 may be regulated by C/EBPβ and associated with anaesthesia‐induced neurotoxicity. A, By double luciferase reporter assay, the transcription factor CCAAT enhancer‐binding protein β (C/EBPβ) interacted with the promoter Rik‐201 and Rik‐203. B, The administration of sevoflurane decreased the mRNA levels of Rik‐201 and Rik‐203. Ctrl, control; Data are represented as mean ± SEM. *P < 0.05, **P < 0.01, ***P < 0.001; by two‐way ANOVA (A); by *t* test (B).For A, n = 3 independent experiments. For B, n = 5 independent experiments

C/EBPβ is a transcription factor that is involved in neurogenesis.^43^ By performing a double luciferase assay, the transcription factor CCAAT enhancer‐binding protein β (C/EBPβ) bounded with the promoter Rik‐201 and Rik‐203, and promoted the transcription (Figure 7A). This suggests that the C/EBPβ may regulate the expression of Rik‐201 and Rik‐203. Additionally, inhaled anaesthetics sevoflurane decreased the levels of Rik‐201 and Rik‐203 in mice brain (Figure 7B). This may further suggested the critical neural‐related regulatory function of Rik‐201 and Rik‐203.

## 4 DISCUSSION

We found that Rik‐201 and Rik‐203 were significantly higher expressed in the mouse brain than in other tissues of E14.5 embryo. This indicted the critical role of Rik‐201 and Rik‐203 functioning as the neural development regulators. A previous study indicated that Rik‐201 (C130071C03Rik) was significantly up‐regulated in mouse glioma, and modulated the gliomagenesis.^29^ LncRNAs function as adaptors, and locate in the nucleus or cytoplasm to regulate the DNA, protein or miRNA functions,^44^, ^45^, ^46^ which indicates their significant central role in the biological process. Additionally, roughly over 50% of LncRNAs express in the central nervous system (CNS).^11^ Rik‐201 and Rik‐203 were up‐regulated during the neural differentiation from mESCs. These results suggested the critical regulatory function of LncRNA‐Rik during the neural differentiation.

LncRNAs have a ceRNA, miRNA sponge‐like, function.^4^, ^42^ Rik‐201 homologous gene ECONEXIN in human glioma cell lines could interact with miR‐411‐5p and modulate the gliomagenesis,^29^ which indicated its ceRNA regulatory function. Rik‐201 and Rik‐203 were located in the cytoplasm. Rik‐201 interacts with miR‐96 and Rik‐203 interacts with miR‐467A‐3p. miR‐96 families have been reported to be the most differentially expressed miRNA between epidermis and neuroectoderm, and could repress neural induction from human embryonic stem cells.^25^ We also found that over‐expression of miR‐96 repressed mouse neural differentiation. miR‐467a‐3p has been reported to be related to breast cancer growth,^47^ and to target some skin‐related genes.^48^ However, there is currently no available data on the neural regulation of the miR‐467a‐3p. We found that over‐expression of miR‐467a‐3p also reduced neural differentiation. Rescue experiments further showed the miR‐96 and miR‐467a‐3p are the downstream targets of Rik‐201 and Rik‐203 during neural differentiation. These results reveal the novel LncRNAs/miRNAs regulatory pathways regulating neural differentiation of NPCs induction from mESCs.

Sox6 was reported to be a critical mediator of maintaining the stemness of the mouse neural stem cells.^49^ Sox6 also forms a positive feedback loop with Sox2, a key transcription factor for NPCs self‐renewal which inhibits neuronal differentiation in the developing central nervous system.^50^ Here, we found that over‐expression of Sox6 restored the mESC neural differentiation to NPCs repressed by knockdown of the Rik‐201 and Rik‐203. Additionally, there are other miRNAs that also target Sox6 in processes such as heart function regulation,^51^ and osteogenic differentiation.^52^ However, there are currently no studies that show the potential impact of miRNA/Sox6 regulatory pathway on the neural development‐related process. Here, we found that both miR‐96 and miR‐467a‐3p targeted the Sox6 to regulate NPCs induction from mESCs. These results indicated the Sox6 being the downstream mediator of LncRNA‐Rik/miRNAs pathway during the neural differentiation. The CCAAT/enhancer‐binding protein β (C/EBPβ) is a key transcription factor implicated in the control of neural differentiation. Mice lacking C/EBPβ present reduced survival of newborn cells in the hippocampus, a decrease in the number of these cells that differentiate into neurons, and a diminished number of cells that proliferate in the subgranular zone of the dentate gyrus.^43^ In this study, it was found that the potential transcriptional activity of C/EBPβ may regulate the expression of Rik‐201 and Rik‐203.

The abnormal neural differentiation leads to further disability of brain.^3^ In mouse embryonic retinas, miR‐96 expression level is minimal and up‐regulates following birth, peaking in adult retinas.^53^ miR‐96 is also reported to target important nerve growth factor family member BDNF^54^, ^55^ that is also involved in the increasing survival of photoreceptors in retinal pigment epithelial (RPE) cells.^56^, ^57^, ^58^ Specific removal of Sox6 results in a severe epileptic encephalopathy.^59^ Downregulation of the Rik‐203 and Rik‐201 releases the miR‐96 to disequilibria the level of miR‐96 during the embryonic neural development. The in vivo experiments also suggested there might be high ratio of mice developmental retardation. This result suggested the potential role of Rik‐203 and Rik‐201 regulating the mice development.

Sevoflurane, the most commonly used general anaesthetic in children, induces neurotoxicity and cognitive impairment in young mice^35^ and inhibits neurogenesis in vitro.^60^, ^61^ The mechanisms by which these effects are created have yet to be identified, impeding further research in anaesthesia neurotoxicity in the developing brain. In December 2016, the FDA issued a warning about the use of general anaesthetics in young children (0‐3 years of age) and pregnant women, and required warnings to be added to the labels of general anaesthetic agents. The widespread and growing use of anaesthesia in children makes its safety a major health issue of interest.^62^ It has become a matter of even greater concern as evidence shows that multiple exposures to anaesthesia and surgery may induce cognitive impairment in children.^63^, ^64^, ^65^, ^66^, ^67^, ^68^ In this study, the administration of sevoflurane decreased the levels of Rik‐201 and Rik‐203. This not only suggests that Rik‐201 and Rik‐203 could be a potential novel target of the anaesthesia neurotoxicity, but also indicated the critical relationship between lncRNA Rik and the neural function.

## CONFLICTS OF INTEREST

The authors declare no competing financial interests.
