# Supplementary figures and images for: LncRNA Riken‐201 and Riken‐203 modulates neural development by regulating the Sox6 through sequestering miRNAs
Source: Cell Prolif. 2019 Jan 22;52(3):e12573. doi: 10.1111/cpr.12573 (PMC6536386; doi:10.1111/cpr.12573)

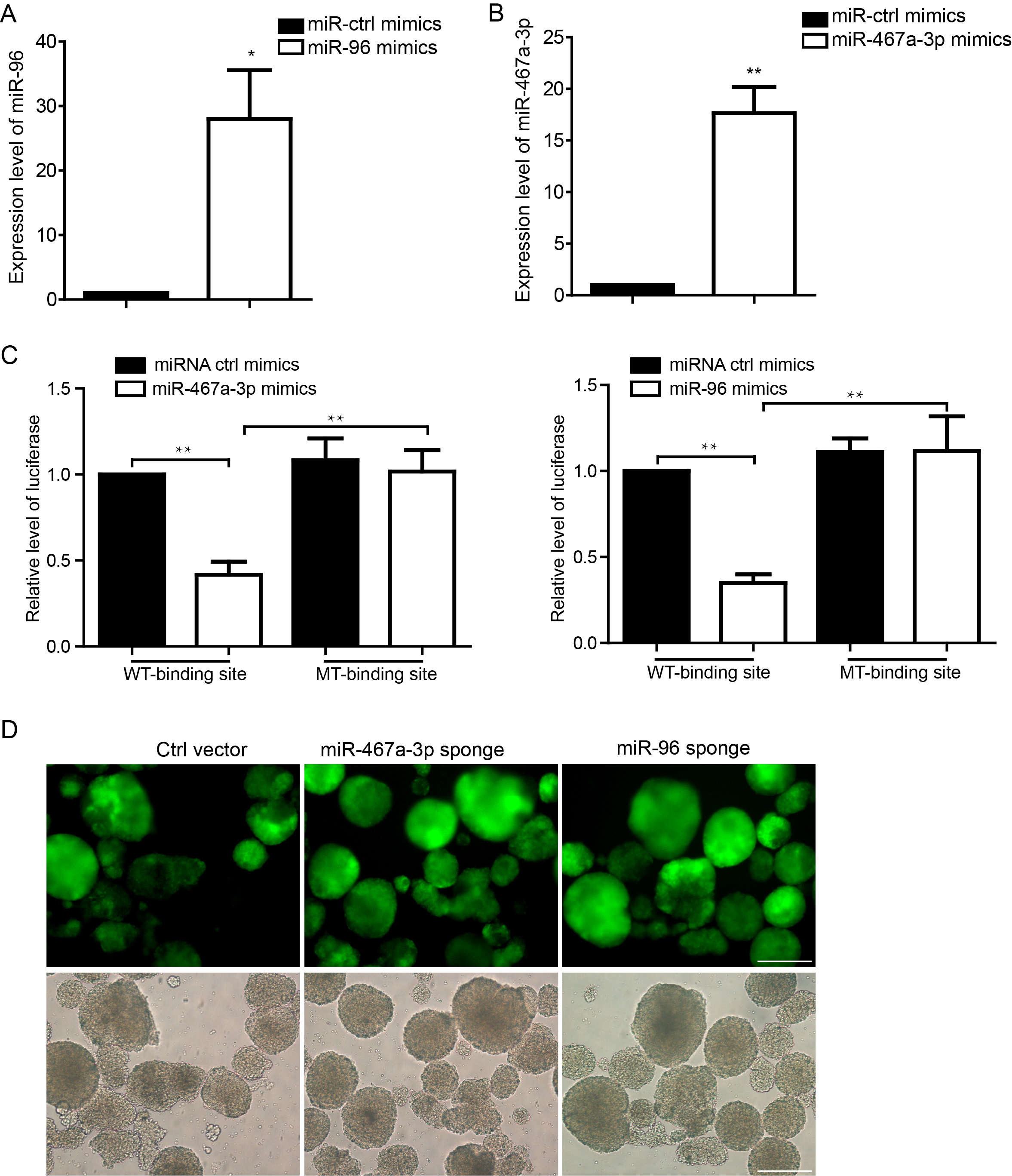

Supplement: Supplementary file 1 [file CPR-52-e12573-s001.tif]

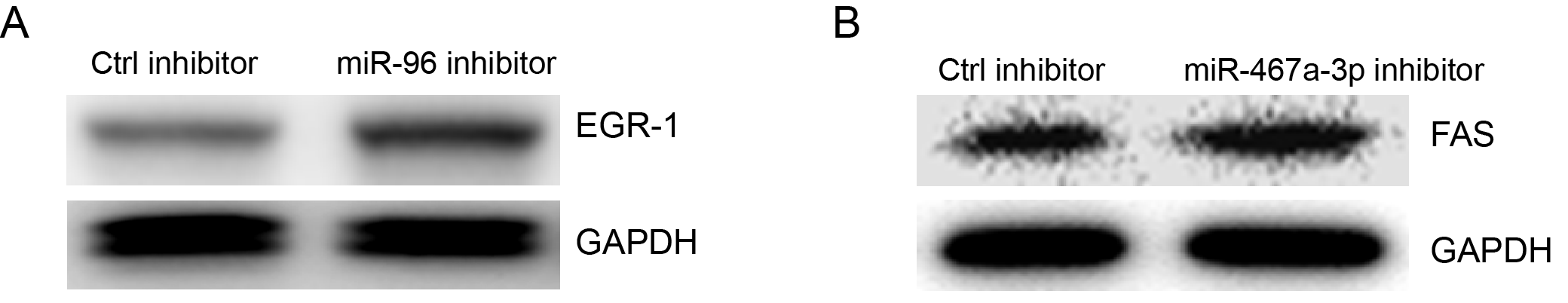

Supplement: Supplementary file 2 [file CPR-52-e12573-s002.tif]

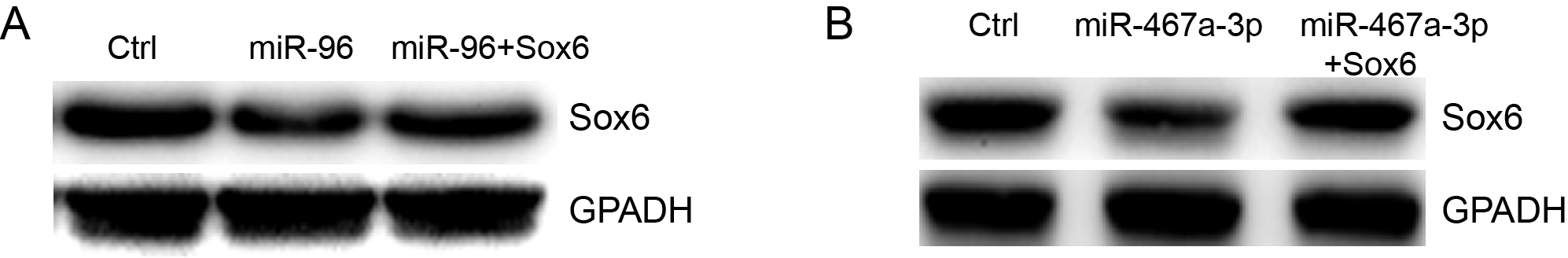

Supplement: Supplementary file 3 [file CPR-52-e12573-s003.tif]
