# Supplementary material for: LncRNA Riken‐201 and Riken‐203 modulates neural development by regulating the Sox6 through sequestering miRNAs
Source: Cell Prolif. 2019 Jan 22;52(3):e12573. doi: 10.1111/cpr.12573 (PMC6536386; doi:10.1111/cpr.12573)
Supplement: Supplementary file 4 [file CPR-52-e12573-s004.docx]

**Supplementary figure legends**

**Supplementary figure 1 related to figure 2. Over-expression of miR-96 and miR-467a-3p via transfecting the synthetic miRNA mimics.**

**A**. miR-96 overexpression via transfecting the synthetic miR-96 mimics during the neural differentiation.**B.** miR-467a-3p overexpression via transfecting the synthetic miR-467a-3p mimics. **C**. Inhibition of miR-467a-3p or miR-96 promotes the efficiency of neural differentiation compared with the empty vector group. miR-ctrl mimics means tansfection of the miRNA mimics control;miR-96 or miR-467a-3p mimics means transfection of the miR-96 or miR-467a-3p miRNA mimics. WT-binding site means the wild type lncRNA sequence luciferase vector. MT-binding site means the mutant type lncRNA sequence luciferase vector .Data are represented as mean ± SEM (n=3).**P*< 0.05, ** *P* <0.01.

**Supplementary figure 2 related to figure3.Western blot detection of the effect of the miR-96 or miR-467a-3p inhibition.**

**A**. Representative pictures of western blot showed that inhibition of miR-96 upregulated the EGR-1 level during the neural differentiation at day 7. **B.** Transfection of the miR-467a-3p inhibitor upregulated the level of Fas expression in 3T3 cells. Ctrl inhibitor means transfection of the miRNA inhibitor control. miR-96or miR-467a-3p inhibitor means transfection of the miR-96 or miR-467a-3p inhibitor control.

**Supplementary figure 3 related to figure4. Overexpression of Sox6 restored miR-96 or miR-467a-3p inducing downregulation level of the protein.**

**A**. Representative pictures of western blot showed that overexpression of Sox6 restored miR-96 inducing downregulation level of the protein. **B**. Overexpression of Sox6 blocked miR-467a-3p inducing downregulation level of protein. Ctrl means miRNA control mimics and empty overexpression vector, miR-96 or miR-467a-3p means miR-96 or miR-467a-3p mimics and empty overexpression vector.
